# The Synergetic Effect of Soft Drinks and Sweet/Salty Snacks Consumption and the Moderating Role of Obesity on Preadolescents’ Emotions and Behavior: A School-Based Epidemiological Study

**DOI:** 10.3390/life13030633

**Published:** 2023-02-24

**Authors:** Ioannis Gketsios, Thomas Tsiampalis, Aikaterini Kanellopoulou, Tonia Vassilakou, Venetia Notara, George Antonogeorgos, Andrea Paola Rojas-Gil, Ekaterina N. Kornilaki, Areti Lagiou, Demosthenes B. Panagiotakos, Rena I. Kosti

**Affiliations:** 1 Department of Nutrition and Dietetics, School of Physical Education, Sports and Dietetics, University of Thessaly, 42132 Trikala, Greece; 2 Department of Nutrition & Dietetics, School of Health Science & Education, Harokopio University, 17671 Athens, Greece; 3 Department of Public Health Policy, School of Public Health, University of West Attica, 11521 Athens, Greece; 4 Laboratory of Hygiene and Epidemiology, Department of Public and Community Health, School of Public Health, University of West Attica, 12243 Athens, Greece; 5 Department of Nursing, Faculty of Health Sciences, University of Peloponnese, 22100 Tripoli, Greece; 6 Department of Preschool Education, School of Education, University of Crete, 74100 Rethimno, Greece; 7 Faculty of Health, University of Canberra, Canberra 2617, Australia

**Keywords:** children, behaviors, emotions, obesity, soft drinks, junk foods

## Abstract

The consumption of ultra-processed foods (UPFs) is high along with the prevalence of emotional and behavioral problems among children and adolescents. The present study sought to investigate the synergetic effect of soft drinks and sweet/salty snacks consumption, and the moderating role of obesity on preadolescents’ emotions and behavior. A cross-sectional study was conducted among 1728 Greek preadolescents aged 10–12 and their parents, during the school years 2014–2016. Parental and child characteristics were collected anonymously, through self-administered and validated questionnaires. Among others, soft drinks and sweet/salty snacks consumption was recorded, classifying preadolescents as low or at least moderate consumers, while anthropometric characteristics (height, weight, Body Mass Index (BMI)) were also recorded. Approximately 6 out of 10 preadolescents were characterized by at least moderate consumption of either sweet/salty snacks, or soft drinks, while 22.7% of the participants had at least moderate consumption of both soft drinks and sweet/salty snacks. Boys and preadolescents with a lower level of adherence to the Mediterranean diet, as well as those living in a more obesogenic family environment were more likely to be in the higher consumption groups. When compared to just either the moderate consumption of sweet/salty snacks, or the moderate consumption of soft drinks, the combination of both unhealthy eating habits was associated with significantly higher odds of both aggressiveness and loneliness, while the examined relationships were significantly stronger in overweight/obese children. The positive synergistic effect of soft drinks and sweet/salty snacks consumption on preadolescents’ emotions of loneliness and aggressive behavior is even burdened by obesity status highlighting the urgent need for policymakers to take preventive measures to halt the detrimental consequences of UPFs consumption on health outcomes, particularly in overweight/obese children. The importance of the improvement of children’s unhealthy eating habits by emphasizing the association between food intake and emotional and behavioral status is highlighted.

## 1. Introduction

Recent evidence suggests that the majority of children and adolescents do not meet the Dietary Guidelines [1], as they prefer to consume ultra–processed foods (UPFs), such as sweet/salty snacks and soft drinks, which are high in added sugar and sodium, low in protein, fiber, and micronutrients [2], instead of adhering to a high-quality diet rich in nutrient-dense foods (e.g., Mediterranean diet) [3]. According to several studies, both the consumption and the availability of UPFs, have reached alarming rates, with their estimated contribution to the total energy intake ranging between 10% and 60% [4,5].Through the displacement of healthy foods in the diet [6], UPFs, tend to cause the deterioration of nutrient profile, which in combination with the satiating properties or the presence of artificial compounds [7], have serious consequences on the prevalence of chronic diseases, such as childhood obesity [8].

Adolescence is a stormy age period in which an “identity crisis” is being faced along with the appearance of a variety of emotional upsets and structural upheavals, while preadolescence or early adolescents (ages 10 to 13) [https://www.healthychildren.org/English/ages-stages/teen/Pages/Stages-of-Adolescence.aspx, accessed on 17 February 2023] constitutes a vulnerable early stage of transition from childhood to adolescence, in which significant physical, emotional, and social changes are being encountered [https://www.ncbi.nlm.nih.gov/books/NBK545476/, accessed on 17 February 2023]. As more than half of mental health problems, such as loneliness and aggression [9,10] begin before the age of 14, the prevention and promotion of mental health at these stages are of paramount importance [11]. Diet quality is considered one of the main determinants of the emotional and psychosocial well-being of children and adolescents [12,13], while at the same time childhood obesity is strongly related to emotional and behavioral problems, as well [14].

Given the association between diet and mental health along with the fact that the majority of youth will experience difficult emotions to some degree during adolescence [15], the present study sought to investigate the effect of soft drink and sweet/salty snack consumption as well as their synergetic effect on loneliness and aggression of Greek preadolescents along with the role of obesity on this association. Of note, recent evidence suggests that the heterogeneous nature of UPFs in nutritional composition imposes the need to be examined as distinct commodities in epidemiological studies as regards their health implications, rather than as a whole group [16].

## 2. Materials and Methods

### 2.1. Design

This is a cross-sectional, school-based, observational study.

### 2.2. Setting

In particular, the study was conducted in various Greek regions, covering approximately 75% of the total population including the greater Athens metropolitan area, the island of Crete and in particular its capital city, Heraklion, as well as the cities of Sparta, Kalamata, and Pyrgos located in the Peloponnese peninsula, during the school years of the period 2014–2016. The scope of sampling was the proportional representation of both urban and rural municipalities of Greece, allowing the generalizability of the results of the study in Greek children of similar age. Both public and private schools were enrolled using random sampling from the list provided by the Greek Ministry of Education and Religious Affairs. All children aged 10–12 were then asked to participate. In total, 47 primary schools (32 from the Athens area, 5 from Heraklion, 3 from Pyrgos, 2 from Kalamata, and 5 from Sparta) were included. More information can be found elsewhere [17].

### 2.3. Sample

In total, 1728 students (795 males; 46%) aged 10–12 years old, enrolled in the study. The participation rate of children ranged from 95% to 100% between schools, without any significant differences between the studied areas. After checking the questionnaires’ completeness for the needs of the present work, the final working sample for the analyses was *n* = 1615 children. All children’s parents were also invited to participate, with a 68.9% response rate being achieved (*n* = 1190).

### 2.4. Bioethics

Before starting the study, approval was requested from the appropriate department of the Ministry of Education and Religious Affairs (code of approval F15/396/72005/C1 by the Institute of Educational Policy) and was carried out following the principles of the Declaration of Helsinki. The investigators informed all people who were involved about the aims and procedures of the research. The students participated in the study after the written consent of their parents.

### 2.5. Measurements

#### 2.5.1. Children’s Characteristics

Each child completed a questionnaire specially developed for the study. Children’s questionnaires were filled in school settings. To avoid errors and discrepancies, the study’s trained investigators assisted children by giving practical examples or solving any doubts when it was necessary. Each child was provided with a personal code by the school principal for the questionnaires to be cross-referenced to those of their parents. The questionnaire retrieved information, among others, about socio-demographic (age, sex), anthropometric characteristics as well as dietary habits, and lifestyle parameters.

#### 2.5.2. Anthropometric Characteristics

Specially trained health scientists/investigators (i.e., dietitians, registered nurses, physicians) took the necessary anthropometric measurements of children (height and weight in cm and kg, respectively) using a tape measure and a scale (with skin-tight clothing, to minimize measurement errors), and performed a face-to-face interview with them, which lasted a maximum of 10–15 min. Each child’s weight (kg) was measured to the nearest 100 gr using a digital scale (Tanita), and height (cm) was measured to the nearest 0.1 cm using a portable stadiometer (Leicester Height Measure). Children’s BMI was calculated as the ratio of kilograms/(height in cm)^2^. Children’s weight status was evaluated through the age- and the sex-specific International Obesity Task Force (IOTF) Body Mass Index cut-off criteria [18].

#### 2.5.3. Physical Activity Status

The standardized, validated, and reliable Physical Activity and Lifestyle Questionnaire (PALQ) [19] was used to measure children’s physical activity status, which was defined as their participation in out-of-school activities such as sports club participation, playing with others, running, and swimming, on a daily or weekly basis.

#### 2.5.4. Level of Adherence to the Mediterranean Diet

The level of adherence to the Mediterranean Diet was evaluated through a special Mediterranean Diet quality index (i.e., KIDMED) [20], in which dietary habits with positive aspect to this dietary pattern scored with +1; dietary habits with negative association scored with −1; and, dietary habits with neutral association scored with 0. The theoretical total score ranges from −4 to 12, with lower scores indicating lower adherence to the Mediterranean diet (≤3, very-low-quality diet; 4–7, need to improve the food pattern to adjust it to the Mediterranean one; ≥8, optimal Mediterranean diet).

#### 2.5.5. Soft Drinks and Sweet/Salty Snacks Consumption

In addition, to acquire information on children’s eating habits, a validated semi-quantitative food frequency questionnaire (FFQ) [21] was also used, which contained all foods and beverages being consumed by the general child population. Based on the children’s responses and following the methodology proposed by Boylan et al. (2017) [22], the sweet/salty snacks intake measure, was based on the consumption of fried potatoes, chocolates/croissants/cookies, and potato crisps/salty snacks. Each food item was assigned a score of 0–5 depending on the frequency of food intake (0 being never/rarely and 5 being 2 or more times per day) so that the sweet/salty snacks intake measure ranged from 0 to 15 (0 being no sweet/salty snacks consumed). Afterwards, based on the median of the sweet/salty snacks intake measure, children were classified as: low sweet/salty snacks consumers (score ≤ 4), and at least moderate sweet/salty snacks consumers (score > 4). Moreover, information regarding the soft drinks’ consumption was also collected, based on daily (1 time or more than 2 times per day), weekly (i.e., 1, 2–6 times per week), or monthly (i.e., 1–3 times per month, less than 1 time per month) basis. Each answer was assigned a score of 0–5 depending on the frequency of soft drinks’ consumption (0 being never/rarely and 5 being 1 or more times per day) so that the soft drinks’ consumption measure ranged from 0 to 5 (0 no soft drinks’ consumption). Afterwards, based on the median of the soft drinks’ consumption, children and were classified as: low soft drinks’ consumers (score ≤ 1), and at least moderate soft drinks’ consumers (score > 1).

#### 2.5.6. Emotions and Behavior

Except for the aforementioned characteristics, participating preadolescents were also asked about the level of aggressiveness and loneliness that they feel. The questions relevant to the emotions and behaviors of children were based on the Adolescent Stress Questionnaire (ASQ) [23] translated and validated in several countries, including Greece [24]. In particular, preadolescents were asked to evaluate how often they feel lonely (never/rarely/sometimes/often/always), as well as how often they argue with their friends and classmates (never/rarely/sometimes/often/always). Based on their responses to the abovementioned questions, preadolescents were classified as having higher level of aggressiveness (sometimes-always vs. never/rarely) and loneliness (sometimes-always vs. never/rarely).

#### 2.5.7. Parental Characteristics

Several parental sociodemographic characteristics (age), anthropometric characteristics [weight (kg) and height (m)], educational level (i.e., primary, secondary, higher), and financial characteristics (income status under or over 18,000 €/year), as well as lifestyle characteristics [smoking status (yes/no), physical activity habit (not at all or at least 1–2 time per week)], were recorded by the children’s parents. The amount of 18,000 euros on family income was set as being the threshold of the average annually income during the period 2014–2016.

#### 2.5.8. Level of Adherence to the Mediterranean Diet

Parents’ level of adherence to the Mediterranean diet was evaluated through a specially designed index, the MedDiet Score (range 0–55), which assesses the level of adherence to the Mediterranean dietary pattern [25]. Higher values on this index indicate greater adherence to this pattern and, consequently, healthier dietary habits. Parents whose score was ≤25 units were classified as being away from the Mediterranean diet, while parents whose score was >25 units were classified as being close/very close to the Mediterranean diet.

### 2.6. Statistical Analysis

Continuous characteristics are presented as mean ± standard deviation (SD), and categorical characteristics as relative frequencies (%). The One-way Analysis of Variance (ANOVA) was used in order to investigate the association between the continuous characteristics and the frequency of junk and soft drinks’ consumption, while the Pearson Chi-square test was used in the case of the categorical characteristics. Normality of the continuous variables’ distribution was tested through graphical (histograms, PP-plots, QQ-plots) and statistical means (Shapiro–Wilk test). Multivariable binomial logistic regression analysis was implemented in order to investigate the association between the aggressiveness (sometimes-always vs. never/rarely) and the loneliness (sometimes-always vs. never/rarely) of the preadolescents, with their frequency of sweet/salty snacks and soft drinks’ consumption. Three separate models were implemented evaluating the effect of the sweet/salty snacks consumption (Model 1), the effect of the soft drinks’ consumption (Model 2), as well as the synergetic effect of the sweet/salty snacks and soft drinks’ consumption (Model 3). Finally, a stratified analysis was performed according to the children’s weight status (normal-weight, overweight/obese). Results are presented as Odds Ratios (OR) and 95% Confidence Intervals (CI), and they are adjusted both for children (age, sex, level of adherence to the Mediterranean diet), as well as their parents’ characteristics (age, sex, BMI, physical activity, education level, income status, and level of adherence to the Mediterranean diet). All statistical analyses were performed using Stata 14.0 (M. Psarros & Assoc., Sparti, Greece) and the significance level was set at a = 0.05.

## 3. Results

### 3.1. Profile of Highsweet/Salty Snacks and Soft Drinks Consuming Preadolescents

Table 1 presents the preadolescents and their parents’ characteristics according to the frequency of sweet/salty snacks and soft drinks’ consumption. Approximately 6 out of 10 preadolescents (59.0%) were characterized by at least moderate consumption of either sweet/salty snacks, or soft drinks, while 22.7% of the participants had at least moderate consumption of both soft drinks and sweet/salty snacks. As depicted, boys (*p* < 0.001), as well as preadolescents with lower level of adherence to the Mediterranean diet (*p* < 0.001) were more likely to be in the higher consumption groups, while as regards their parents’ characteristics, higher soft drinks and sweet/salty snacks consuming preadolescents were more likely to live in more obesogenic family environment, as their parents were characterized by higher body mass index (father: *p* = 0.034; mother: *p* = 0.077 < 0.10). Finally, higher soft drinks and sweet/salty snacks consuming preadolescents seemed to face significantly more problems in terms of their emotional health and behavior, as higher consumption of both soft drinks and sweet/salty snacks were related to significantly higher percentages of both aggressiveness (*p* = 0.002) and loneliness (*p* < 0.001).

### 3.2. Impact of High Sweet/Salty Snacks and Soft Drinks Consumption on Preadolescents’ Levels of Aggressiveness

After adjusting for several preadolescents and their parents’ characteristics, as presented in Table 2, higher sweet/salty snacks and soft drinks consumption was associated with significantly higher odds of being aggressive. More specifically, preadolescents with at least moderate consumption of soft drinks were found to have by 44% (OR = 1.44; 95% CI = 1.15–1.81) higher odds of being aggressive, while preadolescents with at least moderate consumption of sweet/salty snacks were found to have by 50% (OR = 1.50; 95% CI = 1.19–1.88) higher odds of being aggressive. When compared to just either the moderate consumption of sweet/salty snacks, or the moderate consumption of soft drinks, the combination of both unhealthy eating habits (at least moderate consumption of both soft drinks and sweet/salty snacks) was associated with significantly higher odds of aggressiveness, as preadolescents having an at least moderate consumption of both, had approximately twice the odds of being aggressive (OR = 1.90; 95% CI = 1.53–2.45). Finally, it is worth noting the fact that, while normal-weight preadolescents consuming both soft drinks and sweet/salty snacks with an at least moderate frequency had by 52% higher odds of being aggressive (OR = 1.52; 95% CI = 1.31–2.03), overweight/obese preadolescents with the same consumption habits, had approximately quadruple odds of being aggressive (OR = 3.75; 95% CI = 2.38–4.81).

### 3.3. Impact of High Sweet/Salty Snacks and Soft Drinks Consumption on Preadolescents’ Levels of Loneliness

As regards the loneliness of the preadolescents, as depicted in Table 2, higher sweet/salty snacks and soft drinks consumption was also associated with significantly higher odds of feeling lonely on a more frequent basis. More specifically, preadolescents with at least moderate consumption of soft drinks were found to have by 81% (OR = 1.81; 95% CI = 1.41–2.32) higher odds of feeling lonelier, while preadolescents with at least moderate consumption of sweet/salty snacks were found to have by 56% (OR = 1.56; 95% CI = 1.20–2.01) higher odds of feeling lonely on a more frequent basis. When compared to just either the moderate consumption of sweet/salty snacks or the moderate consumption of soft drinks, the combination of both unhealthy eating habits (at least moderate consumption of both soft drinks and sweet/salty snacks) was associated also with significantly higher odds of feeling lonely on a more frequent basis, as preadolescents having at least moderate consumption of both, had at least 2 times higher odds of feeling lonelier (OR = 2.36; 95% CI = 1.88–3.25). Finally, as in the case of the aggressiveness levels, the examined relationship was found to be even stronger among overweight/obese preadolescents, as normal-weight preadolescents consuming both soft drinks and sweet/salty snacks with an at least moderate frequency had by 92% higher odds of feeling lonelier (OR = 1.92; 95% CI = 1.44–2.35), while overweight/obese preadolescents with the same consumption habits, had approximately quadruple odds of being aggressive (OR = 3.70; 95% CI = 2.58–6.61).

## 4. Discussion

To the best of our knowledge, the effect of sweet/salty snacks and soft drinks as well as their synergistic effect on preadolescents’ emotions of loneliness and aggressiveness has rarely been investigated along with the role of obesity in this association. In the present study, it was revealed that nearly 1 out 4 preadolescents are at least moderate consumers of both soft drinks and sweet/salty snacks with the boys, those having unhealthier dietary habits as well as the preadolescents belonging to more obesogenic families, being more likely to consume sweet/salty snacks and soft drinks at least two times per day. Moreover, results showed that the higher the frequency of sweet/salty snacks and soft drinks consumption, the higher the likelihood of preadolescents being afflicted by loneliness and aggression. In particular, it was found that the synergistic effect of consumption is significantly related to even higher odds of feeling lonely and behaving aggressively, with the association being more pronounced among overweight/obese preadolescents.

According to the findings of the 2009/2010 survey of the Health Behavior in Scholl-aged Children (HBSC) the consumption of soft drinks has risen across the globe showing also positive associations with fast-food consumption [https://www.euro.who.int/__data/assets/pdf_file/0006/167424/E96444_part2_4.pdf, accessed on 17 February 2023]. Moreover, a recent report from HBSC study showed that adolescent mental well-being declined in many countries between 2014 and 2018 [https://www.who.int/europe/news/item/19-05-2020-who-report-on-health-behaviours-of-11-15-year-olds-in-europe-reveals-more-adolescents-are-reporting-mental-health-concerns, accessed on 17 February 2023].

Both soft drinks and sweet/salty snacks belong to UPFs which according to NOVA classification, are “made mostly or entirely from substances derived from foods and additives, with little if any intact edible parts of plants or animals minimally modified/preserved” [26]. As anticipated, and in accordance with the global trends, results showed that Greek preadolescents follow unhealthy dietary habits [1,27,28], whereas boys were found to be more susceptible to soft drinks and sweet/salty snacks compared to girls [29,30] perhaps due to the difference in response to environmental food cues, and in particular in high energy density foods [31]. The role of familial aggregation of unhealthy dietary habits is also re-confirmed, highlighting the effect of the family environment in the emotional/relational context [32], as well as the impact of aggressive marketing [33] addressed to youths. Moreover, the association between salt consumption and soft drink consumption has been reported demonstrating salt as the major determinant of fluid and sugar-sweetened soft drink consumption during childhood [34].

In line with the literature, our findings showed that the higher the frequency of sweet/salty snacks and soft drinks consumption, the higher the likelihood of preadolescents being afflicted by loneliness and aggression. Particularly, cross-sectional [35,36] and longitudinal studies [37] suggest a positive association between soft drinks consumption among adolescents and the expression of both aggressive behaviors [38] and loneliness [39,40]. However, in another cross-sectional study consisting of a sample of 13,486 children and adolescents, results showed a significant association between aggression and intake of sweetened beverages and snacks but sweets [41]. The synergistic effect of both soft drinks and sweet/salty snacks on emotional and behavioral problems has been reported in another cross-sectional study conducted in children and adolescents showing that the overall dietary behavior is closely related to emotional health as a result of their higher food additives content [42].

Possible explanations for the observed associations may be attributed to several factors. A recently conducted review in both animal and human studies has shown that excessive consumption of sugar or high palatable foods may cause neurochemical and neurobiological changes, modifying behaviors through the alteration of emotional processing [43]. Furthermore, randomized clinical trials demonstrated the role of micronutrients in emotional regulation and social behavior [44,45,46], underlying the important role of a balanced supply of all essential nutrients through diet on emotional and behavioral well-being. Last but not least, a common characteristic of the overwhelming majority UPFs is their high content of chemicals either in the form of preservatives, coloring, flavoring, stabilizing ingredients, or substances in food contact materials. Several studies have demonstrated that these compounds may act as endocrine disruptors inducing adverse health outcomes [47]. Children and preadolescents due to their higher relative exposures compared with adults show higher susceptibility to the effects of these compounds as a result either due to their immature metabolic system to perform detoxifications processes [48] or through the alteration of gut microbiota [49].

Moreover, in the present study and in accordance with the literature findings the emotional “toll” of excess weight in preadolescents has been observed. The association between the consumption of soft drinks and sweet/salty snacks, and the prevalence of loneliness and aggressive behavior in preadolescents is even more burdened and pronounced among overweight/obese children. The perceived social isolation by preadolescents expressed either as loneliness or aggression as a result of social stigmatization disturbing peer relationships has been reported in the literature [50,51].

To the best of our knowledge, this is the first study in Greece evaluating the synergetic effect of soft drinks and sweet/salty snacks consumption on preadolescents’ emotions and behavior, with the emphasis given on the sub-population of overweight/obese preadolescents. However, given our study’s cross-sectional design, numerous limitations should be addressed, such as the reporting bias introduced by the self-reported questionnaires. To eliminate this form of bias, specially trained investigators were present during the entire process in schools, so as to clarify any potential misunderstandings. Moreover, the present findings cannot be generalized to the entire children’s population but only focused on those aged 10–12 years. Furthermore, due to the observational nature of the study, no temporal relationship and, hence, causal inferences can be made.

## 5. Conclusions

In the present study, it was revealed that the positive synergistic effect of soft and sweet/salty snacks consumption on preadolescents’ emotion of loneliness and aggressive behavior is even burdened by obesity status, highlighting the urgent need for policymakers to take preventive measures to halt the detrimental consequences of UPFs consumption on health particularly in overweight/obese children. It is of utmost importance to communicate to parents about the necessity of the improvement of their children’s unhealthy eating habits by emphasizing the association between food intake and emotional and behavioral status.

## Figures and Tables

**Table 1:** Preadolescents and their parents’ characteristics according to the frequency of their sweet/salty snacks and soft drinks consumption.

	Total Sample (*n* = 1615)	Low Soft Drinks-LOW Sweet/Salty Snacks Consumption (*n* = 663)	LOW Soft Drinks-At Least Moderate Sweet/Salty Snacks Consumption (*n* = 401)	At Least Moderate Soft Drinks-Low Sweet/Salty Snacksconsumption (*n* = 185)	At Least Moderate Soft Drinks-At Least Moderate Sweet/Salty Snacks Consumption (*n* = 366)	*p*-Value
**Children’s characteristics**						
**Age** [in years; Mean (SD)]	11.2 (0.8)	11.1 (0.8)	11.2 (0.8)	11.2 (0.8)	11.4 (0.8)	0.568
**Sex** (Ν (%) Boys)	740 (45.8)	265 (40.0)	168 (41.8)	84 (45.5)	195 (53.4)	**<0.001**
**Body Mass Index** [BMI in kg/m^2^; Mean (SD)]	19.2 (3.4)	19.3 (3.5)	19 (3.3)	19.2 (3.4)	19.4 (3.5)	0.196
**Level of adherence to the Mediterranean diet** (Ν (%) Very low-quality diet)	523 (32.4)	172 (26.0)	130 (32.4)	67 (36.4)	145 (39.7)	**<0.001**
**Parents’ characteristics**						
**Father’s age** [in years; Mean (SD)]	45.8 (5.2)	45.6 (4.9)	46.1 (5.4)	45.9 (5.3)	45.9 (5.3)	0.664
**Mother’s age** [in years; Mean (SD)]	41.5 (4.4)	41.5 (4.2)	41.6 (4.4)	41.7 (4.8)	41.5 (4.6)	0.982
**Father’s educational level** (Ν (%) Higher education)	648 (40.1)	287 (43.3)	170 (42.5)	74 (39.9)	131 (35.8)	0.203
**Mother’s educational level** (Ν (%) Higher education)	730 (45.2)	324 (48.8)	188 (47)	86 (46.3)	152 (41.6)	0.470
**Income status** (Ν (%) > 18,000 euros/year)	819 (50.7)	351 (52.9)	204 (50.9)	92 (49.5)	126 (34.3)	0.190
**Father’s Body Mass Index** [in kg/m^2^; Mean (SD)]	27 (3.7)	27.2 (3.8)	26.7 (3.4)	25.6 (4.1)	27.2 (3.8)	**0.034**
**Mother’s Body Mass Index** [in kg/m^2^; Mean (SD)]	24 (4.0)	24.2 (4.2)	23.7 (3.8)	22.9 (4.1)	24.2 (3.9)	**0.077**
**Smoking habits** (Ν (%) At least one parent smokes)	861 (53.3)	333 (50.3)	205 (51.2)	97 (52.3)	214 (58.5)	0.147
**Level of adherence to the Mediterranean diet** (Ν (%) Away from the Mediterranean diet)	811 (50.2)	267 (40.2)	201 (50.2)	100 (54.2)	227 (61.9)	**<0.001**
**Mental health-Behaviour problems**						
**Aggressiveness** (Ν (%) Sometimes/Always)	530 (32.8)	178 (26.8)	136 (33.8)	67 (36.2)	140 (38.3)	**0.002**
**Loneliness** (Ν (%) Sometimes/Always)	323 (20)	103 (15.5)	73 (18.1)	42 (22.6)	98 (26.9)	**<0.001**

Notes: *p*-value was based on the One-way Analysis of Variance (ANOVA) in case of continuous characteristics and Pearson Chi-square test in case of categorical characteristics; SD = Standard Deviation; BMI = Body Mass Index; Parental level of adherence to the Mediterranean diet was estimated through the MedDietScore, and those scoring ≤ 25 units, were classified as being Away from the Mediterranean diet; KIDMED score ranges from −4 to 12-Lower scores indicate low adherence to the Mediterranean diet while higher scores, high adherence to the Mediterranean diet (≤3, very-low-quality diet; 4–7, need to improve the food pattern to adjust it to the Mediterranean one; ≥ 8, optimal Mediterranean diet).

**Table 2:** Results from binomial logistic regression analysis evaluating the association of soft drinks and sweet/salty snacks consumption with the preadolescents’ likelihood of feeling anger and loneliness at a more frequent basis, both in the total sample, as well as stratified by their weight status.

OR (95% CI)	Aggressiveness		
	Total Sample	Normal-Weight Children	Overweight/Obese Children
**Model 1: Soft drinks consumption (Ref: Low soft drinks consumption)**			
*At least moderate soft drinks consumption*	1.44 (1.15, 1.81) **	1.28 (0.97, 1.48) *	1.99 (1.31, 3.04) **
**Model 2: Sweet/salty snacks consumption (Ref: Low sweet/salty snacks consumption)**			
*At least moderate sweet/salty snacks consumption*	1.50 (1.19, 1.88) ***	1.27 (0.97, 1.47) *	2.36 (1.51, 3.69) ***
**Model 3: Soft drinks + sweet/salty snacks consumption (Ref: Low soft drinks + sweet/salty snacks consumption)**			
*Low soft drinks + At least moderate sweet/salty snacks consumption*	1.40 (1.06, 1.85) **	1.22 (0.88, 1.71)	2.08 (1.21, 3.58) **
*At least moderate soft drinks + Low sweet/salty snacks consumption*	1.55 (0.83, 2.90)	1.41 (0.69, 2.87)	2.22 (0.59, 8.35)
*At least moderate soft drinks + At least moderate sweet/salty snacks consumption*	1.90 (1.53, 2.45) ***	1.52 (1.31, 2.03) **	3.75 (2.38, 4.81) ***
	**Loneliness**		
**Model 1: Soft drinks consumption (Ref: Low soft drinks consumption)**			
*At least moderate soft drinks consumption*	1.81 (1.41, 2.32) ***	1.40 (1.03, 1.89) **	2.55 (1.15, 3.23) ***
**Model 2: Sweet/salty snacks consumption (Ref: Low sweet/salty snacks consumption)**			
*At least moderate sweet/salty snacks consumption*	1.56 (1.20, 2.01) ***	1.29 (0.95, 1.75)	2.39 (1.22, 3.47) **
**Model 3: Soft drinks + sweet/salty snacks food consumption (Ref: Low soft drinks + sweet/salty snacks consumption)**			
*Low soft drinks + At least moderate sweet/salty snacks consumption*	1.21 (0.87, 1.67)	1.17 (0.80, 1.72)	1.33 (0.68, 2.59)
*At least moderate soft drinks + Low sweet/salty snacks consumption*	1.60 (0.81, 3.16)	1.49 (0.74, 3.34)	1.73 (0.34, 8.71)
*At least moderate soft drinks + At least moderate sweet/salty snacks consumption*	2.36 (1.88, 3.25) ***	1.92 (1.44, 2.35) **	3.70 (2.58, 6.61) ***

Notes: OR = Odds Ratio; CI = Confidence Interval; Results are adjusted for preadolescents (age, sex, level of adherence to the Mediterranean diet) and their parents’ (age, sex, BMI, physical activity, education level, income status, and level of adherence to the Mediterranean diet) characteristics. *** *p* < 0.001; ** *p* < 0.05; * *p* < 0.10.

## Data Availability

Data are available upon request. The data are not publicly available due to privacy or ethical restrictions.
